# Poly[[bis­[μ_2_-*N*,*N*′-bis­(2-pyridyl­meth­yl)oxalamide-κ^4^ *N*,*O*:*N*′,*O*′][μ_2_-*N*,*N*′-bis­(2-pyridyl­meth­yl)oxalamide-κ^2^ *N*:*N*′]disilver(I)] bis­(trifluoro­methane­sulfonate)]

**DOI:** 10.1107/S1600536810033611

**Published:** 2010-08-28

**Authors:** Hadi D. Arman, Tyler Miller, Pavel Poplaukhin, Edward R. T. Tiekink

**Affiliations:** a Department of Chemistry, The University of Texas at San Antonio, One UTSA Circle, San Antonio, Texas 78249-0698, USA; b Chemical Abstracts Service, 2540 Olentangy River Rd, Columbus, Ohio, 43202, USA; c Department of Chemistry, University of Malaya, 50603 Kuala Lumpur, Malaysia

## Abstract

The asymmetric unit of the title salt, [Ag(C_14_H_14_N_4_O_2_)_1.5_](CF_3_SO_3_), comprises a Ag^+^ cation, three half-mol­ecules of *N*,*N*′-bis­(2-pyridyl­meth­yl)oxalamide (each of which is dis­posed about a centre of inversion) and a trifluoro­methane­sulfonate anion. Distinct coordination modes are found for the bridging ligands, *i.e*., a μ_2_,κ^2^-bridging mode involving pyridine N atoms for one ligand, and a μ_2_,κ^4^-bridging mode, employing both pyridine N and amide O atoms for the remaining ligands. The Ag^+^ cations, which are in a distorted square-pyramidal coordination, and the ligands combine to form a two-dimensional array parallel to (101); these arrays are connected into a three-dimensional structure by trifluoro­methane­sulfonate anions *via* N—H⋯O, C—H⋯O, and C—F⋯O inter­actions.

## Related literature

For structural diversity in the structures of silver salts, see: Kundu *et al.* (2010 ▶). For crystal engineering studies on isomeric *N*,*N*′-bis­(3-pyridyl­meth­yl)oxalamides, see: Poplaukhin & Tiekink (2010 ▶). For the structure of the BF_4_^−^ salt, see: Schauer *et al.* (1998 ▶). For additional structural analysis, see: Addison *et al.* (1984 ▶).
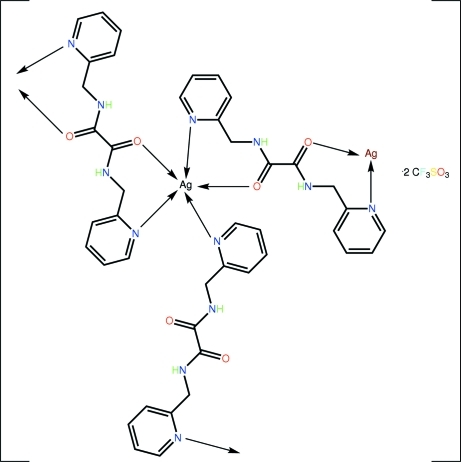


## Experimental

### 

#### Crystal data

[Ag(C_14_H_14_N_4_O_2_)_1.5_](CF_3_SO_3_)
*M* *_r_* = 662.38
Triclinic, 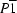

*a* = 8.7242 (14) Å
*b* = 11.1762 (17) Å
*c* = 14.210 (2) Å
α = 95.977 (1)°
β = 105.948 (2)°
γ = 107.017 (3)°
*V* = 1247.9 (3) Å^3^
*Z* = 2
Mo *K*α radiation
μ = 0.97 mm^−1^
*T* = 98 K
0.36 × 0.32 × 0.18 mm

#### Data collection

Rigaku AFC12/SATURN724 diffractometer
Absorption correction: multi-scan (*ABSCOR*; Higashi, 1995 ▶) *T*_min_ = 0.868, *T*_max_ = 1.000
10177 measured reflections
5688 independent reflections
5438 reflections with *I* > 2σ(*I*)
*R*_int_ = 0.029

#### Refinement

*R*[*F* ^2^ > 2σ(*F* ^2^)] = 0.034
*wR*(*F* ^2^) = 0.079
*S* = 1.05
5688 reflections
352 parameters
H-atom parameters constrained
Δρ_max_ = 0.67 e Å^−3^
Δρ_min_ = −1.05 e Å^−3^

### 

Data collection: *CrystalClear* (Molecular Structure Corporation & Rigaku, 2005 ▶); cell refinement: *CrystalClear*; data reduction: *CrystalClear*; program(s) used to solve structure: *SHELXS97* (Sheldrick, 2008 ▶); program(s) used to refine structure: *SHELXL97* (Sheldrick, 2008 ▶); molecular graphics: *ORTEP-3* (Farrugia, 1997 ▶) and *DIAMOND* (Brandenburg, 2006 ▶); software used to prepare material for publication: *publCIF* (Westrip, 2010 ▶).

## Supplementary Material

Crystal structure: contains datablocks global, I. DOI: 10.1107/S1600536810033611/pv2322sup1.cif

Structure factors: contains datablocks I. DOI: 10.1107/S1600536810033611/pv2322Isup2.hkl

Additional supplementary materials:  crystallographic information; 3D view; checkCIF report

## Figures and Tables

**Table 1:** Selected bond lengths (Å)

Ag—N1	2.378 (2)
Ag—N3	2.210 (2)
Ag—N5	2.250 (2)
Ag—O1	2.9665 (19)
Ag—O2	2.7299 (17)

**Table 2:** Hydrogen-bond geometry (Å, °)

*D*—H⋯*A*	*D*—H	H⋯*A*	*D*⋯*A*	*D*—H⋯*A*
N2—H2n⋯O4^i^	0.88	2.17	2.992 (4)	156
N4—H4n⋯O3^ii^	0.88	2.19	2.980 (3)	149
N6—H6n⋯O6^iii^	0.88	2.22	2.936 (3)	139
C1—H1⋯O5^iv^	0.95	2.36	3.197 (3)	146
C17—H17⋯O4	0.95	2.43	3.334 (4)	158
C18—H18⋯F1	0.95	2.45	3.261 (4)	144
N2—H2n⋯O1^v^	0.88	2.32	2.697 (3)	106
N4—H4n⋯O2^ii^	0.88	2.32	2.692 (3)	105
N6—H6n⋯O3^vi^	0.88	2.34	2.709 (3)	106

Symmetry codes: (i) 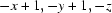; (ii) 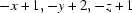; (iii) 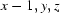; (iv) 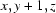; (v) 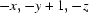; (vi) 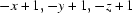.

**Fig. 1.**
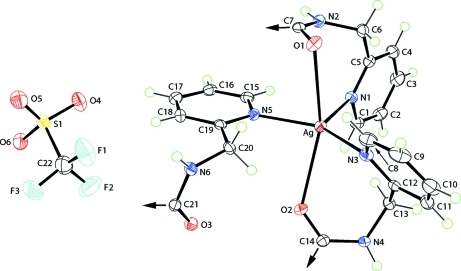
An asymmetric unit of (I) showing the atom-labelling scheme and displacement ellipsoids at the 50% probability level. Each of the N,N'-bis(2-pyridylmethyl)oxalamide molecules is situated about a centre of inversion.

**Fig. 2.**
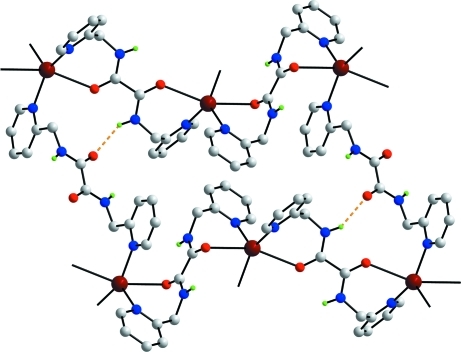
A part of the 2-D grid in (I) showing the µ2 and µ4– (twice) modes of coordination of the N,N'-bis(2-pyridylmethyl)oxalamide ligands. The N–H···O hydrogen bonds are shown as orange dashed lines. The trifluoromethanesulfonate anions and the C-bound hydrogen atoms have been omitted for clarity.

**Fig. 3.**
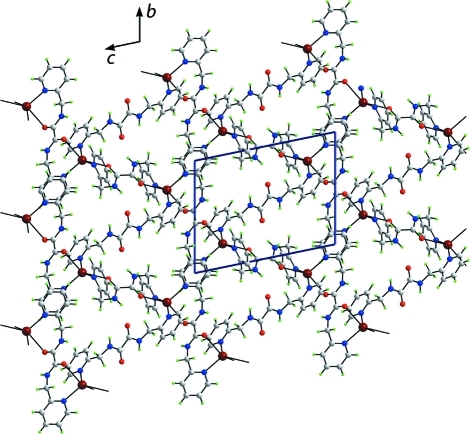
A view in projection down the a axis of the 2-D grid in (I). The trifluoromethanesulfonate anions have been omitted for clarity.

**Fig. 4.**
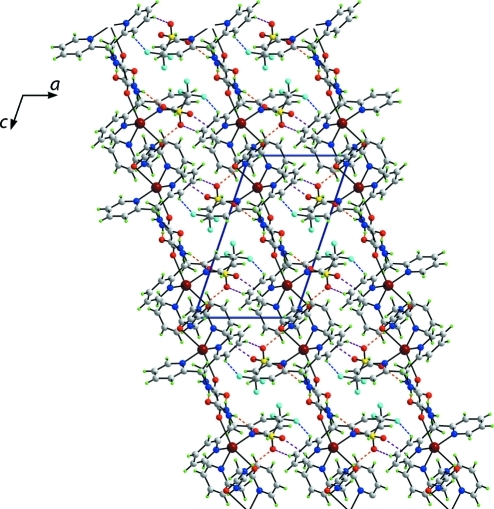
A view in projection down the b axis of the crystal packing in (I). The layers shown in Fig. 3 are interspersed by the trifluoromethanesulfonate anions which are connected by N–H···O hydrogen bonds (orange dashed lines) to one layer, and C–H···O and C–H···F interactions (shown as purple and blue dashed lines, respectively) to the other.

## supplementary crystallographic information

### Comment

For silver salts, the dependence of crystal structure upon counter anions and the presence of solvent is notorious and has ramifications for photoluminescence (Kundu *et al.*, 2010). In connection with crystal engineering studies on the isomeric *N*,*N*'-bis(*n*-pyridylmethyl)oxalamides (Poplaukhin & Tiekink, 2010), the 3:2 reaction between Ag(trifluoromethanesulfonate) and *N*,*N*'-bis(2-pyridylmethyl)oxalamide in an ethanol/chloroform solution was investigated, which led to the characterization of the title compound, (I).

The asymmetric unit of (I) comprises a Ag cation, three half molecules of *N*,*N*'-bis(2-pyridylmethyl)oxalamide (each of which is disposed about a centre of inversion) and a trifluoromethanesulfonate anion. Each of the ligands coordinates to a Ag atom, one employing the pyridine-N atoms exclusively while the others are µ_2_,κ^4^-bridging, employing both pyridine-N and amide-O atoms, leading to non-planar seven-membered chelate rings, Fig. 2. It is noted that the Ag–O bond distances are significantly longer than the Ag–N bond distances, Table 1. The resulting N_3_O_2_ coordination geometry is distorted square pyramidal based on the value for τ in (I) of 0.02 compared to the ideal values for τ of 0.0 and 1.0 for ideal square pyramidal and trigonal bi-pyramidal geometries, respectively (Addison *et al.*, 1984). In this description, the Ag atoms lies 0.7272 (10) Å out of the plane defined by the O1,O2,N3 and N5 atoms (r.m.s. deviation = 0.0805 Å) in the direction of the N1 atom.

The 2-D structure observed for (I) contrasts the helical coordination polymer observed in the structure of the silver tetrafluoroborate salt containing the same ligand, isolated as a hydrate (Schauer *et al.*, 1998). The *N*,*N*'-bis(2-pyridylmethyl)oxalamide ligand acts as a bidentate donor employing both pyridine-N atoms in coordination (Schauer *et al.*, 1998).

The crystal packing in (I) can be envisaged as chains of Ag atoms bridged by the µ_2_,κ^4^-ligands linked by the µ_2_,κ^2^-ligands leading to 2-D arrays parallel to (1 0 1), Fig. 3. The layers are connected by contacts involving the trifluoromethanesulfonate anions. Thus, the trifluoromethanesulfonate anions participate in N–H···O hydrogen bonds formed to one layer, and C–H···O and C–H···F interactions to the other, Fig. 4 and Table 2. In addition to the intermolecular interactions, intramolecular N–H···O hydrogen bonds are also noted, Table 2.

### Experimental

Colourless crystals of (I) were isolated from the 3:2 reaction of Ag(trifluoromethanesulfonate) (Sigma-Aldrich, 0.06 mmol) and *N*,*N*'-bis(2-pyridylmethyl)oxalamide (0.04 mmol) in a warm ethanol/chloroform solution (8 ml).

### Refinement

C-bound H-atoms were placed in calculated positions (N–H = 0.88 Å and C–H 0.95–0.99 Å) and were included in the refinement in the riding model approximation with *U*_iso_(H) set to 1.2*U*_eq_(C). The maximum and minimum residual electron density peaks of 0.67 and -1.05 e Å^-3^, respectively, were located 0.85 Å and 0.79 Å from the S1 and Ag atoms, respectively.

### Crystal data

**Table:**

[Ag(C_14_H_14_N_4_O_2_)_1.5_](CF_3_SO_3_)	*Z* = 2
*M**_r_* = 662.38	*F*(000) = 666
Triclinic, *P*1	*D*_x_ = 1.763 Mg m^−^^3^
Hall symbol: -P 1	Mo *K*α radiation, λ = 0.71069 Å
*a* = 8.7242 (14) Å	Cell parameters from 5470 reflections
*b* = 11.1762 (17) Å	θ = 2.7–40.5°
*c* = 14.210 (2) Å	µ = 0.97 mm^−^^1^
α = 95.977 (1)°	*T* = 98 K
β = 105.948 (2)°	Plate, colourless
γ = 107.017 (3)°	0.36 × 0.32 × 0.18 mm
*V* = 1247.9 (3) Å^3^	

### Data collection

**Table:**

Rigaku AFC12K/SATURN724 diffractometer	5688 independent reflections
Radiation source: fine-focus sealed tube	5438 reflections with *I* > 2σ(*I*)
graphite	*R*_int_ = 0.029
ω scans	θ_max_ = 27.5°, θ_min_ = 2.0°
Absorption correction: multi-scan (*ABSCOR*; Higashi, 1995)	*h* = −11→11
*T*_min_ = 0.868, *T*_max_ = 1.000	*k* = −14→14
10177 measured reflections	*l* = −18→10

### Refinement

**Table:**

Refinement on *F*^2^	Primary atom site location: structure-invariant direct methods
Least-squares matrix: full	Secondary atom site location: difference Fourier map
*R*[*F*^2^ > 2σ(*F*^2^)] = 0.034	Hydrogen site location: inferred from neighbouring sites
*wR*(*F*^2^) = 0.079	H-atom parameters constrained
*S* = 1.05	*w* = 1/[σ^2^(*F*_o_^2^) + (0.0295*P*)^2^ + 1.3977*P*] where *P* = (*F*_o_^2^ + 2*F*_c_^2^)/3
5688 reflections	(Δ/σ)_max_ = 0.001
352 parameters	Δρ_max_ = 0.67 e Å^−^^3^
0 restraints	Δρ_min_ = −1.05 e Å^−^^3^

### Special details

**Table:**

Geometry. All e.s.d.'s (except the e.s.d. in the dihedral angle between two l.s. planes) are estimated using the full covariance matrix. The cell e.s.d.'s are taken into account individually in the estimation of e.s.d.'s in distances, angles and torsion angles; correlations between e.s.d.'s in cell parameters are only used when they are defined by crystal symmetry. An approximate (isotropic) treatment of cell e.s.d.'s is used for estimating e.s.d.'s involving l.s. planes.
Refinement. Refinement of *F*^2^ against ALL reflections. The weighted *R*-factor *wR* and goodness of fit *S* are based on *F*^2^, conventional *R*-factors *R* are based on *F*, with *F* set to zero for negative *F*^2^. The threshold expression of *F*^2^ > σ(*F*^2^) is used only for calculating *R*-factors(gt) *etc*. and is not relevant to the choice of reflections for refinement. *R*-factors based on *F*^2^ are statistically about twice as large as those based on *F*, and *R*- factors based on ALL data will be even larger.

### Fractional atomic coordinates and isotropic or equivalent isotropic displacement parameters (Å^2^)

**Table:**

	*x*	*y*	*z*	*U*_iso_*/*U*_eq_	
Ag	0.18992 (2)	0.789941 (17)	0.198313 (13)	0.01680 (6)	
S1	0.83484 (8)	0.26442 (7)	0.26813 (5)	0.02348 (14)	
F1	0.6446 (3)	0.3310 (3)	0.36252 (17)	0.0586 (6)	
F2	0.8906 (4)	0.4736 (2)	0.39309 (18)	0.0663 (7)	
F3	0.8647 (3)	0.3038 (2)	0.45763 (14)	0.0527 (6)	
O1	−0.1034 (2)	0.56970 (17)	0.06781 (14)	0.0230 (4)	
O2	0.4189 (2)	0.89384 (17)	0.38477 (13)	0.0210 (4)	
O3	0.5809 (2)	0.65495 (16)	0.47965 (13)	0.0180 (3)	
O4	0.7817 (3)	0.3298 (2)	0.18909 (16)	0.0330 (5)	
O5	0.7255 (2)	0.1362 (2)	0.25641 (17)	0.0346 (5)	
O6	1.0141 (2)	0.28246 (18)	0.29925 (16)	0.0284 (4)	
N1	0.2257 (3)	0.8994 (2)	0.06692 (15)	0.0171 (4)	
N2	0.0110 (3)	0.63965 (19)	−0.05116 (15)	0.0183 (4)	
H2N	0.0695	0.6247	−0.0898	0.022*	
N3	0.0373 (3)	0.8513 (2)	0.28181 (15)	0.0178 (4)	
N4	0.3734 (3)	1.07931 (19)	0.42923 (15)	0.0162 (4)	
H4N	0.4058	1.1475	0.4765	0.019*	
N5	0.3388 (2)	0.65910 (18)	0.18415 (15)	0.0148 (4)	
N6	0.3291 (3)	0.49849 (19)	0.39599 (15)	0.0166 (4)	
H6N	0.2627	0.4214	0.3942	0.020*	
C1	0.3585 (3)	1.0079 (2)	0.09336 (19)	0.0203 (5)	
H1	0.4419	1.0246	0.1568	0.024*	
C2	0.3804 (3)	1.0972 (2)	0.0328 (2)	0.0229 (5)	
H2	0.4757	1.1735	0.0545	0.028*	
C3	0.2598 (4)	1.0721 (3)	−0.0599 (2)	0.0257 (6)	
H3	0.2707	1.1308	−0.1035	0.031*	
C4	0.1235 (3)	0.9603 (2)	−0.08807 (19)	0.0219 (5)	
H4	0.0394	0.9414	−0.1516	0.026*	
C5	0.1091 (3)	0.8756 (2)	−0.02376 (18)	0.0177 (5)	
C6	−0.0395 (3)	0.7529 (2)	−0.0529 (2)	0.0210 (5)	
H6A	−0.1056	0.7548	−0.0066	0.025*	
H6B	−0.1140	0.7473	−0.1210	0.025*	
C7	−0.0292 (3)	0.5578 (2)	0.00759 (18)	0.0168 (5)	
C8	−0.1050 (3)	0.7645 (3)	0.2844 (2)	0.0240 (5)	
H8	−0.1325	0.6781	0.2532	0.029*	
C9	−0.2128 (3)	0.7952 (3)	0.3302 (2)	0.0292 (6)	
H9	−0.3111	0.7310	0.3314	0.035*	
C10	−0.1752 (3)	0.9210 (3)	0.3744 (2)	0.0286 (6)	
H10	−0.2494	0.9452	0.4042	0.034*	
C11	−0.0268 (3)	1.0114 (3)	0.37424 (19)	0.0224 (5)	
H11	0.0028	1.0983	0.4049	0.027*	
C12	0.0774 (3)	0.9732 (2)	0.32879 (17)	0.0159 (4)	
C13	0.2458 (3)	1.0686 (2)	0.33435 (17)	0.0173 (5)	
H13A	0.2820	1.0402	0.2783	0.021*	
H13B	0.2335	1.1531	0.3286	0.021*	
C14	0.4416 (3)	0.9886 (2)	0.44598 (17)	0.0155 (4)	
C15	0.4180 (3)	0.6709 (2)	0.11494 (17)	0.0180 (5)	
H15	0.4053	0.7318	0.0741	0.022*	
C16	0.5166 (3)	0.5990 (2)	0.10053 (18)	0.0203 (5)	
H16	0.5752	0.6132	0.0532	0.024*	
C17	0.5288 (3)	0.5057 (3)	0.15634 (19)	0.0237 (5)	
H17	0.5921	0.4522	0.1462	0.028*	
C18	0.4466 (3)	0.4921 (2)	0.22738 (19)	0.0207 (5)	
H18	0.4524	0.4284	0.2663	0.025*	
C19	0.3555 (3)	0.5721 (2)	0.24127 (17)	0.0147 (4)	
C20	0.2721 (3)	0.5694 (2)	0.32159 (17)	0.0167 (5)	
H20A	0.2964	0.6582	0.3552	0.020*	
H20B	0.1480	0.5304	0.2900	0.020*	
C21	0.4798 (3)	0.5475 (2)	0.46690 (17)	0.0152 (4)	
C22	0.8089 (5)	0.3483 (3)	0.3762 (2)	0.0387 (7)	

### Atomic displacement parameters (Å^2^)

**Table:**

	*U*^11^	*U*^22^	*U*^33^	*U*^12^	*U*^13^	*U*^23^
Ag	0.01925 (10)	0.01673 (10)	0.01533 (10)	0.00778 (7)	0.00530 (7)	0.00263 (7)
S1	0.0203 (3)	0.0251 (3)	0.0249 (3)	0.0084 (3)	0.0054 (3)	0.0072 (3)
F1	0.0680 (15)	0.0967 (19)	0.0516 (13)	0.0604 (15)	0.0387 (12)	0.0368 (13)
F2	0.108 (2)	0.0355 (12)	0.0527 (14)	0.0256 (13)	0.0241 (14)	−0.0027 (10)
F3	0.0741 (15)	0.0691 (15)	0.0260 (9)	0.0405 (13)	0.0131 (10)	0.0164 (10)
O1	0.0281 (9)	0.0181 (9)	0.0243 (9)	0.0069 (7)	0.0121 (8)	0.0028 (7)
O2	0.0221 (9)	0.0180 (9)	0.0183 (8)	0.0072 (7)	0.0015 (7)	−0.0030 (7)
O3	0.0194 (8)	0.0137 (8)	0.0183 (8)	0.0030 (7)	0.0046 (7)	0.0043 (7)
O4	0.0376 (11)	0.0423 (12)	0.0318 (11)	0.0238 (10)	0.0157 (9)	0.0182 (10)
O5	0.0208 (9)	0.0291 (11)	0.0438 (12)	0.0022 (8)	0.0010 (9)	0.0082 (10)
O6	0.0176 (9)	0.0223 (10)	0.0402 (11)	0.0034 (7)	0.0049 (8)	0.0058 (9)
N1	0.0180 (10)	0.0182 (10)	0.0151 (9)	0.0065 (8)	0.0046 (8)	0.0040 (8)
N2	0.0201 (10)	0.0135 (9)	0.0195 (10)	0.0045 (8)	0.0050 (8)	0.0033 (8)
N3	0.0151 (9)	0.0199 (10)	0.0169 (10)	0.0047 (8)	0.0042 (8)	0.0032 (8)
N4	0.0167 (9)	0.0143 (9)	0.0140 (9)	0.0046 (8)	0.0014 (8)	−0.0005 (8)
N5	0.0152 (9)	0.0131 (9)	0.0156 (9)	0.0043 (7)	0.0047 (8)	0.0022 (7)
N6	0.0174 (9)	0.0128 (9)	0.0171 (9)	0.0024 (8)	0.0032 (8)	0.0064 (8)
C1	0.0179 (11)	0.0223 (12)	0.0186 (11)	0.0054 (10)	0.0054 (10)	0.0008 (10)
C2	0.0238 (13)	0.0166 (12)	0.0259 (13)	0.0015 (10)	0.0108 (11)	0.0017 (10)
C3	0.0358 (15)	0.0180 (12)	0.0259 (13)	0.0079 (11)	0.0132 (12)	0.0100 (10)
C4	0.0273 (13)	0.0203 (12)	0.0176 (12)	0.0095 (10)	0.0040 (10)	0.0058 (10)
C5	0.0193 (11)	0.0152 (11)	0.0183 (11)	0.0063 (9)	0.0053 (9)	0.0025 (9)
C6	0.0193 (12)	0.0157 (12)	0.0244 (12)	0.0062 (10)	0.0014 (10)	0.0039 (10)
C7	0.0148 (10)	0.0133 (11)	0.0160 (11)	0.0020 (9)	−0.0005 (9)	−0.0006 (9)
C8	0.0180 (12)	0.0254 (13)	0.0216 (12)	0.0010 (10)	0.0031 (10)	0.0031 (10)
C9	0.0160 (12)	0.0426 (17)	0.0222 (13)	0.0006 (11)	0.0061 (10)	0.0052 (12)
C10	0.0214 (13)	0.0506 (18)	0.0181 (12)	0.0169 (13)	0.0080 (11)	0.0062 (12)
C11	0.0231 (12)	0.0309 (14)	0.0174 (11)	0.0156 (11)	0.0059 (10)	0.0050 (10)
C12	0.0168 (11)	0.0197 (12)	0.0108 (10)	0.0079 (9)	0.0015 (9)	0.0038 (9)
C13	0.0194 (11)	0.0176 (11)	0.0156 (11)	0.0078 (9)	0.0043 (9)	0.0054 (9)
C14	0.0127 (10)	0.0153 (11)	0.0166 (11)	0.0025 (8)	0.0048 (9)	0.0013 (9)
C15	0.0203 (11)	0.0176 (11)	0.0131 (10)	0.0041 (9)	0.0034 (9)	0.0025 (9)
C16	0.0195 (11)	0.0222 (12)	0.0162 (11)	0.0056 (10)	0.0053 (9)	−0.0027 (9)
C17	0.0256 (13)	0.0245 (13)	0.0201 (12)	0.0127 (11)	0.0039 (10)	−0.0025 (10)
C18	0.0249 (12)	0.0183 (12)	0.0191 (12)	0.0119 (10)	0.0027 (10)	0.0035 (10)
C19	0.0129 (10)	0.0132 (10)	0.0133 (10)	0.0020 (8)	0.0000 (8)	0.0010 (8)
C20	0.0176 (11)	0.0164 (11)	0.0153 (11)	0.0061 (9)	0.0031 (9)	0.0052 (9)
C21	0.0190 (11)	0.0151 (11)	0.0135 (10)	0.0071 (9)	0.0065 (9)	0.0038 (9)
C22	0.0490 (19)	0.0446 (19)	0.0307 (16)	0.0252 (16)	0.0144 (15)	0.0101 (14)

### Geometric parameters (Å, °)

**Table:**

Ag—N1	2.378 (2)	C2—H2	0.9500
Ag—N3	2.210 (2)	C3—C4	1.378 (4)
Ag—N5	2.250 (2)	C3—H3	0.9500
Ag—O1	2.9665 (19)	C4—C5	1.386 (3)
Ag—O2	2.7299 (17)	C4—H4	0.9500
S1—O5	1.433 (2)	C5—C6	1.510 (3)
S1—O4	1.447 (2)	C6—H6A	0.9900
S1—O6	1.4487 (19)	C6—H6B	0.9900
S1—C22	1.818 (3)	C7—C7^i^	1.537 (5)
F1—C22	1.344 (4)	C8—C9	1.381 (4)
F2—C22	1.333 (4)	C8—H8	0.9500
F3—C22	1.333 (4)	C9—C10	1.382 (4)
O1—C7	1.225 (3)	C9—H9	0.9500
O2—C14	1.228 (3)	C10—C11	1.391 (4)
O3—C21	1.225 (3)	C10—H10	0.9500
N1—C1	1.341 (3)	C11—C12	1.385 (3)
N1—C5	1.347 (3)	C11—H11	0.9500
N2—C7	1.337 (3)	C12—C13	1.518 (3)
N2—C6	1.457 (3)	C13—H13A	0.9900
N2—H2N	0.8800	C13—H13B	0.9900
N3—C8	1.346 (3)	C14—C14^ii^	1.538 (5)
N3—C12	1.352 (3)	C15—C16	1.376 (4)
N4—C14	1.329 (3)	C15—H15	0.9500
N4—C13	1.461 (3)	C16—C17	1.386 (4)
N4—H4N	0.8800	C16—H16	0.9500
N5—C19	1.344 (3)	C17—C18	1.387 (4)
N5—C15	1.345 (3)	C17—H17	0.9500
N6—C21	1.334 (3)	C18—C19	1.392 (3)
N6—C20	1.452 (3)	C18—H18	0.9500
N6—H6N	0.8800	C19—C20	1.511 (3)
C1—C2	1.390 (4)	C20—H20A	0.9900
C1—H1	0.9500	C20—H20B	0.9900
C2—C3	1.382 (4)	C21—C21^iii^	1.543 (4)
			
N3—Ag—N5	145.64 (8)	O1—C7—N2	125.9 (2)
N3—Ag—N1	114.52 (7)	O1—C7—C7^i^	121.7 (3)
N5—Ag—N1	99.84 (7)	N2—C7—C7^i^	112.3 (3)
N3—Ag—O2	77.39 (7)	N3—C8—C9	123.0 (3)
N5—Ag—O2	86.55 (6)	N3—C8—H8	118.5
N1—Ag—O2	117.91 (6)	C9—C8—H8	118.5
N3—Ag—O1	92.93 (6)	C8—C9—C10	118.9 (3)
N5—Ag—O1	84.19 (6)	C8—C9—H9	120.5
N1—Ag—O1	94.90 (6)	C10—C9—H9	120.5
O2—Ag—O1	146.99 (5)	C9—C10—C11	118.8 (2)
O5—S1—O4	115.07 (13)	C9—C10—H10	120.6
O5—S1—O6	115.22 (12)	C11—C10—H10	120.6
O4—S1—O6	114.01 (13)	C12—C11—C10	119.1 (3)
O5—S1—C22	102.86 (16)	C12—C11—H11	120.4
O4—S1—C22	104.09 (14)	C10—C11—H11	120.4
O6—S1—C22	103.39 (15)	N3—C12—C11	122.2 (2)
C7—O1—Ag	91.70 (14)	N3—C12—C13	118.0 (2)
C14—O2—Ag	131.58 (16)	C11—C12—C13	119.8 (2)
C1—N1—C5	117.9 (2)	N4—C13—C12	109.80 (19)
C1—N1—Ag	115.41 (16)	N4—C13—H13A	109.7
C5—N1—Ag	125.33 (16)	C12—C13—H13A	109.7
C7—N2—C6	121.8 (2)	N4—C13—H13B	109.7
C7—N2—H2N	119.1	C12—C13—H13B	109.7
C6—N2—H2N	119.1	H13A—C13—H13B	108.2
C8—N3—C12	117.9 (2)	O2—C14—N4	126.2 (2)
C8—N3—Ag	118.87 (18)	O2—C14—C14^ii^	120.5 (3)
C12—N3—Ag	123.19 (16)	N4—C14—C14^ii^	113.3 (3)
C14—N4—C13	121.6 (2)	N5—C15—C16	123.1 (2)
C14—N4—H4N	119.2	N5—C15—H15	118.5
C13—N4—H4N	119.2	C16—C15—H15	118.5
C19—N5—C15	118.4 (2)	C15—C16—C17	118.8 (2)
C19—N5—Ag	124.21 (15)	C15—C16—H16	120.6
C15—N5—Ag	117.34 (16)	C17—C16—H16	120.6
C21—N6—C20	121.6 (2)	C16—C17—C18	118.5 (2)
C21—N6—H6N	119.2	C16—C17—H17	120.7
C20—N6—H6N	119.2	C18—C17—H17	120.7
N1—C1—C2	123.4 (2)	C17—C18—C19	119.6 (2)
N1—C1—H1	118.3	C17—C18—H18	120.2
C2—C1—H1	118.3	C19—C18—H18	120.2
C3—C2—C1	118.2 (2)	N5—C19—C18	121.4 (2)
C3—C2—H2	120.9	N5—C19—C20	115.7 (2)
C1—C2—H2	120.9	C18—C19—C20	122.9 (2)
C4—C3—C2	118.8 (2)	N6—C20—C19	113.0 (2)
C4—C3—H3	120.6	N6—C20—H20A	109.0
C2—C3—H3	120.6	C19—C20—H20A	109.0
C3—C4—C5	120.0 (2)	N6—C20—H20B	109.0
C3—C4—H4	120.0	C19—C20—H20B	109.0
C5—C4—H4	120.0	H20A—C20—H20B	107.8
N1—C5—C4	121.7 (2)	O3—C21—N6	125.6 (2)
N1—C5—C6	117.5 (2)	O3—C21—C21^iii^	121.4 (3)
C4—C5—C6	120.9 (2)	N6—C21—C21^iii^	113.0 (2)
N2—C6—C5	113.0 (2)	F3—C22—F2	109.1 (3)
N2—C6—H6A	109.0	F3—C22—F1	106.6 (3)
C5—C6—H6A	109.0	F2—C22—F1	107.8 (3)
N2—C6—H6B	109.0	F3—C22—S1	111.0 (2)
C5—C6—H6B	109.0	F2—C22—S1	111.4 (2)
H6A—C6—H6B	107.8	F1—C22—S1	110.8 (2)
			
N3—Ag—O1—C7	163.63 (15)	Ag—O1—C7—C7^i^	111.3 (3)
N5—Ag—O1—C7	−50.71 (15)	C6—N2—C7—O1	−3.3 (4)
N1—Ag—O1—C7	48.71 (15)	C6—N2—C7—C7^i^	176.9 (2)
O2—Ag—O1—C7	−125.18 (15)	C12—N3—C8—C9	1.8 (4)
N3—Ag—O2—C14	−39.2 (2)	Ag—N3—C8—C9	−176.6 (2)
N5—Ag—O2—C14	171.4 (2)	N3—C8—C9—C10	1.0 (4)
N1—Ag—O2—C14	72.1 (2)	C8—C9—C10—C11	−2.4 (4)
O1—Ag—O2—C14	−114.8 (2)	C9—C10—C11—C12	0.9 (4)
N3—Ag—N1—C1	87.98 (19)	C8—N3—C12—C11	−3.3 (3)
N5—Ag—N1—C1	−91.61 (18)	Ag—N3—C12—C11	175.01 (18)
O2—Ag—N1—C1	−0.3 (2)	C8—N3—C12—C13	174.4 (2)
O1—Ag—N1—C1	−176.54 (17)	Ag—N3—C12—C13	−7.3 (3)
N3—Ag—N1—C5	−78.4 (2)	C10—C11—C12—N3	2.0 (4)
N5—Ag—N1—C5	102.0 (2)	C10—C11—C12—C13	−175.7 (2)
O2—Ag—N1—C5	−166.73 (18)	C14—N4—C13—C12	74.8 (3)
O1—Ag—N1—C5	17.0 (2)	N3—C12—C13—N4	−95.2 (2)
N5—Ag—N3—C8	−60.0 (2)	C11—C12—C13—N4	82.5 (3)
N1—Ag—N3—C8	120.70 (19)	Ag—O2—C14—N4	−11.8 (4)
O2—Ag—N3—C8	−124.1 (2)	Ag—O2—C14—C14^ii^	168.21 (18)
O1—Ag—N3—C8	23.97 (19)	C13—N4—C14—O2	6.2 (4)
N5—Ag—N3—C12	121.69 (19)	C13—N4—C14—C14^ii^	−173.8 (2)
N1—Ag—N3—C12	−57.6 (2)	C19—N5—C15—C16	−0.6 (3)
O2—Ag—N3—C12	57.57 (18)	Ag—N5—C15—C16	178.29 (18)
O1—Ag—N3—C12	−154.33 (18)	N5—C15—C16—C17	3.2 (4)
N3—Ag—N5—C19	2.5 (3)	C15—C16—C17—C18	−2.6 (4)
N1—Ag—N5—C19	−178.21 (18)	C16—C17—C18—C19	−0.4 (4)
O2—Ag—N5—C19	64.05 (18)	C15—N5—C19—C18	−2.5 (3)
O1—Ag—N5—C19	−84.22 (18)	Ag—N5—C19—C18	178.64 (17)
N3—Ag—N5—C15	−176.41 (15)	C15—N5—C19—C20	176.4 (2)
N1—Ag—N5—C15	2.93 (18)	Ag—N5—C19—C20	−2.4 (3)
O2—Ag—N5—C15	−114.81 (17)	C17—C18—C19—N5	3.0 (4)
O1—Ag—N5—C15	96.91 (17)	C17—C18—C19—C20	−175.8 (2)
C5—N1—C1—C2	0.9 (4)	C21—N6—C20—C19	76.1 (3)
Ag—N1—C1—C2	−166.6 (2)	N5—C19—C20—N6	−165.2 (2)
N1—C1—C2—C3	−0.7 (4)	C18—C19—C20—N6	13.7 (3)
C1—C2—C3—C4	0.2 (4)	C20—N6—C21—O3	2.8 (4)
C2—C3—C4—C5	0.1 (4)	C20—N6—C21—C21^iii^	−175.8 (2)
C1—N1—C5—C4	−0.6 (4)	O5—S1—C22—F3	64.0 (3)
Ag—N1—C5—C4	165.49 (18)	O4—S1—C22—F3	−175.7 (2)
C1—N1—C5—C6	179.8 (2)	O6—S1—C22—F3	−56.3 (3)
Ag—N1—C5—C6	−14.1 (3)	O5—S1—C22—F2	−174.2 (2)
C3—C4—C5—N1	0.2 (4)	O4—S1—C22—F2	−53.9 (3)
C3—C4—C5—C6	179.8 (2)	O6—S1—C22—F2	65.5 (3)
C7—N2—C6—C5	120.3 (2)	O5—S1—C22—F1	−54.3 (3)
N1—C5—C6—N2	−58.2 (3)	O4—S1—C22—F1	66.1 (3)
C4—C5—C6—N2	122.2 (3)	O6—S1—C22—F1	−174.5 (2)
Ag—O1—C7—N2	−68.5 (2)		

Symmetry codes: (i) −*x*, −*y*+1, −*z*; (ii) −*x*+1, −*y*+2, −*z*+1; (iii) −*x*+1, −*y*+1, −*z*+1.

### Hydrogen-bond geometry (Å, °)

**Table:**

*D*—H···*A*	*D*—H	H···*A*	*D*···*A*	*D*—H···*A*
N2—H2n···O4^iv^	0.88	2.17	2.992 (4)	156
N4—H4n···O3^ii^	0.88	2.19	2.980 (3)	149
N6—H6n···O6^v^	0.88	2.22	2.936 (3)	139
C1—H1···O5^vi^	0.95	2.36	3.197 (3)	146
C17—H17···O4	0.95	2.43	3.334 (4)	158
C18—H18···F1	0.95	2.45	3.261 (4)	144
N2—H2n···O1^i^	0.88	2.32	2.697 (3)	106
N4—H4n···O2^ii^	0.88	2.32	2.692 (3)	105
N6—H6n···O3^iii^	0.88	2.34	2.709 (3)	106

Symmetry codes: (iv) −*x*+1, −*y*+1, −*z*; (ii) −*x*+1, −*y*+2, −*z*+1; (v) *x*−1, *y*, *z*; (vi) *x*, *y*+1, *z*;  (i) −*x*, −*y*+1, −*z*; (iii) −*x*+1, −*y*+1, −*z*+1.
